# The Relationship Between Health Literacy Level and Media Used as a Source of Health-Related Information

**DOI:** 10.3928/24748307-20210330-01

**Published:** 2021-04

**Authors:** Seçil Özkan, Hakan Tüzün, Asiye Uğraş Dikmen, Nur Baran Aksakal, Deniz Çalışkan, Özge Taşçı, Selime Ceylan Güneş

## Abstract

**Background:**

Previous studies have not shown the level of health literacy or associated factors on a national level in Turkey using a scale that has been adapted to the country and its culture.

**Objective:**

This study aimed to determine health literacy levels in Turkey and to investigate the association of health literacy with socioeconomic factors as well as with the instruments used as sources of health-related information.

**Methods:**

This cross-sectional, nationally representative study was conducted using a computer-assisted personal interview approach and included 6,228 households (response rate, 70.9%). The Turkey Health Literacy Scale was used to measure health literacy. Sources of health-related information, such as newspapers, television, internet, and smartphones, were included in the regression model for health literacy.

**Key Results:**

The proportion of participants with inadequate and problematic health literacy was 30.9% and 38%, respectively, showing that approximately 7 of 10 participants had limited health literacy. The frequencies of inadequate and problematic health literacy were higher in the disease prevention and promotion domains (37.4% and 34.2%, respectively) compared with those in the health care domain (27.1% and 31.3%, respectively). The most frequently used medium as a source of health-related information was the internet (48.6%), followed by television (33%). In controlled models, higher health literacy scores were associated with higher education and income levels. The effects of television (β = 1,917), internet (β = 2,803), newspapers (β = 1,489), and smartphones (β = 1,974) as sources of health-related information were statistically significant in the general health literacy index model.

**Conclusions:**

Health literacy in Turkey reflects social inequalities. The model accounting for socioeconomic variables demonstrated the relevance of sources of health information to level of health literacy. These findings emphasize the importance of improving sources of health information to improve health literacy. **[*HLRP: Health Literacy Research and Practice*. 2021;5(2):e109–e117.]**

**Plain Language Summary:**

This is a cross-sectional study that is representative of the population of Turkey. We reported that health literacy scores were higher for people in higher levels of socioeconomic status. We showed that using the television, internet, newspapers, and smartphones as a source of health-related information is associated with health literacy even when accounting for socioeconomic variables.

Health literacy (HL) has drawn the attention of researchers and policymakers as a concept that is associated with certain key elements, such as health determinants, health outputs, and health behavior (Kickbusch et al., 2013). Limited HL is a global health issue. In the United States, for example, the proportions of adults with basic and below basic HL were 22% and 14%, respectively (Kutner et al., 2006). In the European health literacy survey (HLS-EU), which was conducted in eight European Union countries, approximately one-half of the participants had limited (insufficient or problematic) HL (Sørensen et al., 2015). Studies conducted in various developing countries worldwide have shown that HL is limited in more than one-half of the relevant populations (Jovic-Vranes et al., 2011; Karimi et al., 2014; Toci et al., 2014; Wu et al., 2017).

The factors that affect HL include age, education, income, and employment status, as well as the level of health knowledge. Adults who are older, groups with lower income, groups with lower education levels, and immigrant and minority groups are among the main risk groups for low HL (Beauchamp et al., 2015; Bo et al., 2014; Liu et al., 2015; Sørensen et al., 2015).

The types of media used as a source of health-related information have been investigated in various studies regarding health-related information-seeking behavior. The prevalence of internet use in seeking health-related information is higher than that of other sources (Swoboda et al., 2018; Wong & Cheung, 2019). On the other hand, previous studies have shown that different sources of health-related information have different levels of impact on HL level. Some studies have shown that the internet has had the greatest impact on HL (Kobayashi et al., 2015; Lubetkin et al., 2015). Another study showed that the effects of the internet, books, and magazines are close (Cutilli et al., 2018). According to another study, smartphones have an impact on HL (Bailey et al., 2015).

Considering the different aspects of HL that are associated with determinants of health, such as gender and education level, HL is a variable that can be more easily changed or improved. However, mechanisms that link HL to disparities in health are not well explored (Mantwill & Diviani, 2019). Investigating how types of media that are used as a source of health-related information and socioeconomic determinants together affect the level of HL may lead to a better understanding of this mechanism.

The educational level in Turkey is lower than that of other developed countries (Eurostat, 2018). This suggests that the proportion of risk groups with low HL may be higher in Turkey. The strategic objectives of the Turkish Ministry of Health include “the development of health literacy to increase individuals' responsibility for their own health,” which indicates that there is an awareness of this issue at the political level in Turkey (Türkiye Cumhuriyeti Sağlik Bakanliği, 2021). To ensure that the actions implemented for this objective achieve their targets, it would be beneficial to identify groups with limited HL in Turkey.

Further studies are necessary to determine the interventions that are required for increasing HL among both health care providers and patients (Barry et al., 2012; Davis & Wolf, 2004). Although the subject of HL has been gaining increasing attention in Turkey in recent years, studies are typically of a local nature or include only certain groups (Atay et al., 2018; Bodur et al., 2017; Caylan et al., 2017; Eyüboğlu & Schulz, 2016).

The scale used in the HLS-EU study consists of three domains: (1) disease prevention, (2) health promotion, and (3) health care (Sørensen et al., 2013). Health prevention and health promotion practices are intertwined in Turkey, especially for primary health care services. Therefore, the “protection of health” and “disease prevention” titles are combined in the Turkey Health Literacy Scale (THLS) (Okyay et al., 2015). The HLS-EU scale consists of four dimensions: (1) accessing, (2) understanding, (3) appraising, and (4) applying health-related information (Sørensen et al., 2013). The THLS also consists of four dimensions, as in the original scale. It is well known that the distribution of HL varies from one culture to another (Mantwill & Diviani, 2019). In the current study, the important factor, HL, was taken into consideration when revising the HLS-EU scale. To the best of our knowledge, the present study is the first to determine HL in Turkey at a national level with a scale that is adapted to the country's conditions and culture.

This study aimed to determine HL levels in Turkey and investigate the association of HL with socioeconomic factors as well as with sources used for obtaining health-related information.

## Methods

### Sampling

This was a cross-sectional study whose design was based on the Nomenclature of Territorial Units for Statistics Level 1 (a geocode standard for referencing the subdivisions of countries for statistical purposes). It was conducted on a sample population that is representative of Turkey in general. The study sample was determined by the Turkish Statistical Institute using the Address-Based Population Registration System database. A stratified three-stage cluster sampling method was used in this study. The calculated sample size was 9,980 households. The sample volume was calculated as 9,980 households by considering the possibility of a 30% loss.

### Data Collection

**Figure 1.**
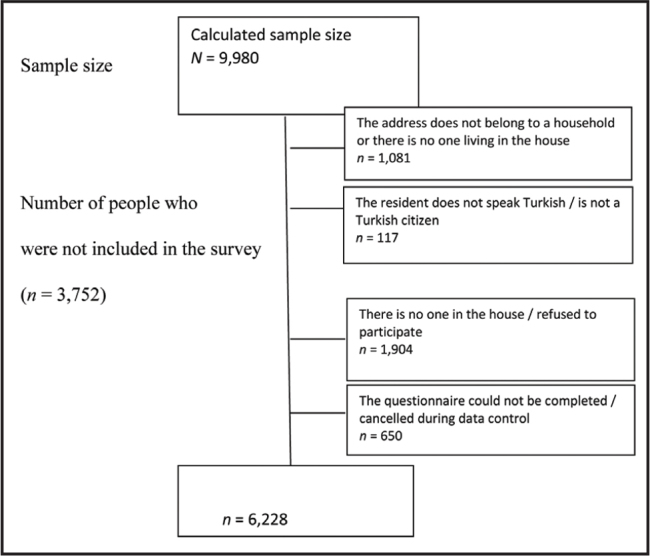
Flow chart of the study.

All residents of Turkey were eligible to participate in the study. In total, the survey was administered to 6,228 households. Addresses that “did not belong to a household or there was no one living in the house” (*n* = 1,081) and in which “the resident did not speak Turkish or was not a Turkish citizen” (*n* = 117) were excluded. The response rate according to the number of houses within the scope (*n* = 8,782) was 70.9% (**Figure 1**).

Data collection was conducted between March 21, 2017 and May 18, 2017. The face-to-face survey was administered using the computer-assisted personal interview approach.

This article was prepared using data from Turkish National Health Literacy Survey of the Turkish Ministry of Health, with the permission of the managers and employees named in the Acknowledgment section who worked in the Turkish General Directorate of Health Promotion during the study.

### Health Literacy Questionnaire

The THLS is a 32-item scale that was adapted from the conceptual model and definition developed by the HLS-EU consortium. However, the THLS considers two relevant domains instead of the three specified in HLS-EU. These two domains are “health care” and “disease prevention and health promotion.” The THLS has four dimensions like the HLS-EU; therefore, the matrix of the THLS has eight cells. Each cell has four items. Possible item responses are *very easy, easy, difficult,* and *very difficult,* as with the HLS-EU. Cronbach's alpha for the THLS scale was determined to be 0.927 (Okyay et al., 2015; Okyay & Abacigil, 2016).

To evaluate the THLS, indices were standardized between 0 and 50 (as in the HLS-EU), and the following formula was used: HL index = (mean − 1) × (50/3).

The general HL index, health care HL index, and disease prevention and health promotion HL index were calculated accordingly.

The resulting index was classified into four categories: inadequate (0–25), problematic (>25–33), sufficient (>33–42), and excellent (>42–50). The inadequate and problematic levels were classified as limited HL.

### Statistical Analysis

Statistical analyses were performed using SPSS (version 22). Data were analyzed in accordance with the multistage sampling method using the complex sample module. Descriptive statistics were expressed as weighted percentages and confidence intervals.

Linear regression analysis was used to analyze the demographic and socioeconomic characteristics associated with HL. The dependent variables were general, health care, disease prevention, and health promotion HL indices. Independent quantitative variables included the age group (18–24, 25–34, 35–44, 45–54, 55–64, and ≥65 years), educational level (illiterate, literate but did not graduate from any school, primary school graduate, secondary school graduate, high school graduate, and university graduate), and household income level, which was evaluated by “adequacy of income in meeting household needs” (quite insufficient, insufficient, neutral, sufficient, and quite sufficient). The independent categorical variables included sex (female/male), having social security (no/yes), using the newspaper as a source of information (no/yes), using the television as a source of health-related information (no/yes), using the internet as a source of health-related information (no/yes), and using a smartphone as a source of health-related information (no/yes). In the regression analysis, for categorical variables, the first categories (e.g., female; no) specified here were the reference variables.

### Ethical Approval

The study was approved by the Ethics Committee of Gazi University in its meeting number 03, held on March 7, 2017 (number E.40835).

## Results

Basic sociodemographic characteristics of the study population are presented in **Table 1**.

**Table 1:**
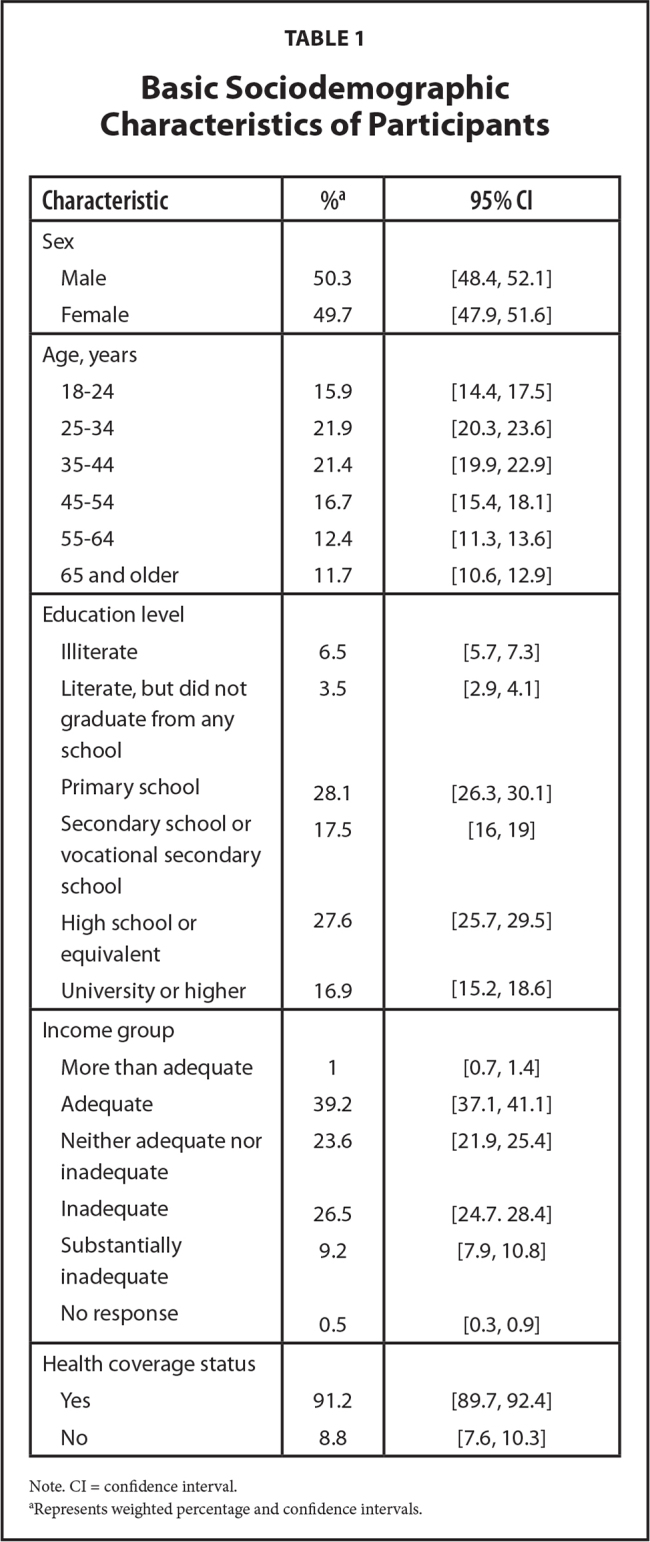
Basic Sociodemographic Characteristics of Participants

**Characteristic**	%**^a^**	**95% CI**
Sex		
Male	50.3	[48.4, 52.1]
Female	49.7	[47.9, 51.6]

Age, years		
18–24	15.9	[14.4, 17.5]
25–34	21.9	[20.3, 23.6]
35–44	21.4	[19.9, 22.9]
45–54	16.7	[15.4, 18.1]
55–64	12.4	[11.3, 13.6]
65 and older	11.7	[10.6, 12.9]

Education level		
Illiterate	6.5	[5.7, 7.3]
Literate, but did not graduate from any school	3.5	[2.9, 4.1]
Primary school	28.1	[26.3, 30.1]
Secondary school or vocational secondary school	17.5	[16, 19]
High school or equivalent	27.6	[25.7, 29.5]
University or higher	16.9	[15.2, 18.6]

Income group		
More than adequate	1	[0.7, 1.4]
Adequate	39.2	[37.1, 41.1]
Neither adequate nor inadequate	23.6	[21.9, 25.4]
Inadequate	26.5	[24.7. 28.4]
Substantially inadequate	9.2	[7.9, 10.8]
No response	0.5	[0.3, 0.9]

Health coverage status		
Yes	91.2	[89.7, 92.4]
No	8.8	[7.6, 10.3]

Note. CI = confidence interval.

a Represents weighted percentage and confidence intervals.

**Figure 2.**
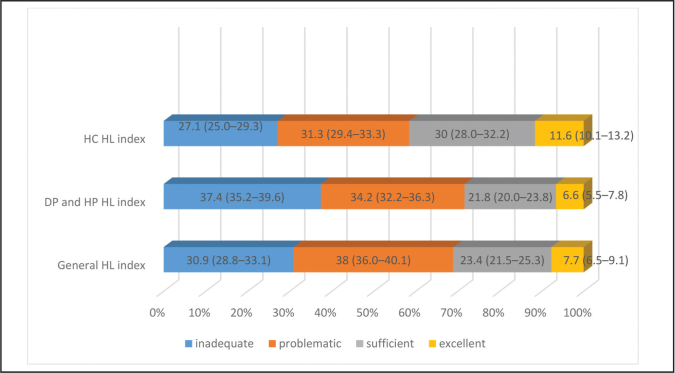
Health literacy levels (95% confidence interval). DP = disease prevention; HL = health literacy; HP = health promotion, HC = health care.

The proportions of people with inadequate and problematic HL were 30.9% and 38%, respectively, indicating that HL is limited in approximately 7 of 10 people. The frequency of inadequate and problematic HL was higher in the disease prevention and promotion domains (37.4% and 34.2%, respectively) compared with that in the health care domain (27.1% and 31.3%, respectively) (**Figure 2**).

Of the respondents, 33% stated that they did not use any medium as a source of health-related information. The most frequently used medium as a source of health-related information was the internet (48.6%), followed by television (33%) (**Table 2**).

**Table 2:**
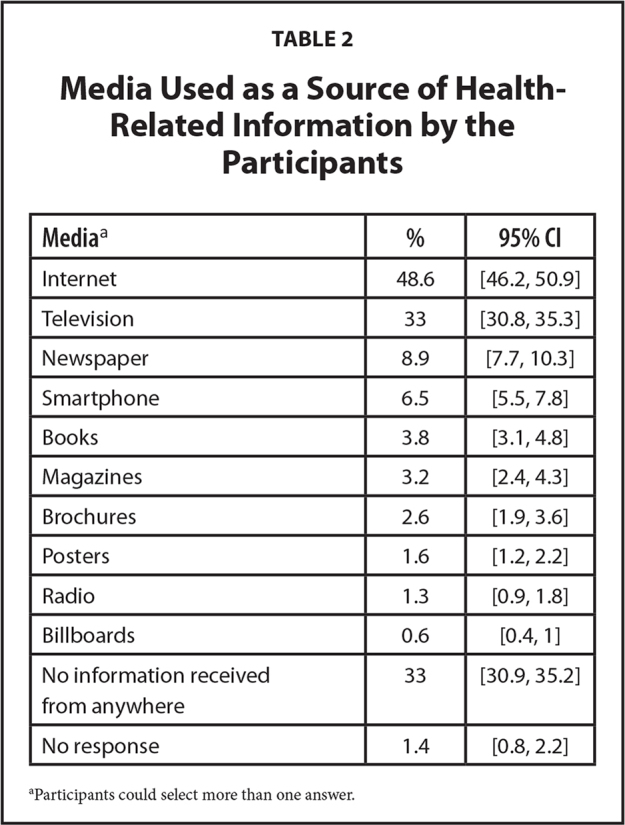
Media Used as a Source of Health-Related Information by the Participants

**Media**^a^	**%**	**95% CI**
Internet	48.6	[46.2, 50.9]
Television	33	[30.8, 35.3]
Newspaper	8.9	[7.7, 10.3]
Smartphone	6.5	[5.5, 7.8]
Books	3.8	[3.1, 4.8]
Magazines	3.2	[2.4, 4.3]
Brochures	2.6	[1.9, 3.6]
Posters	1.6	[1.2, 2.2]
Radio	1.3	[0.9, 1.8]
Billboards	0.6	[0.4, 1]
No information received from anywhere	33	[30.9, 35.2]
No response	1.4	[0.8, 2.2]

a Participants could select more than one answer.

**Table 3** shows the linear regression models created for the HL scores. The score decreased with age in all models. Sex had a statistically significant effect in the model for the general and health care HL indices, and men had higher levels of HL. In all of the models, higher HL scores were associated with higher educational level and income, and they were higher among people who reported that they used television, the internet, newspapers, and smartphones as a source of health-related information. However, for the health care HL index, the use of newspapers as a source of health-related information did not show a considerable effect.

**Table 3:**
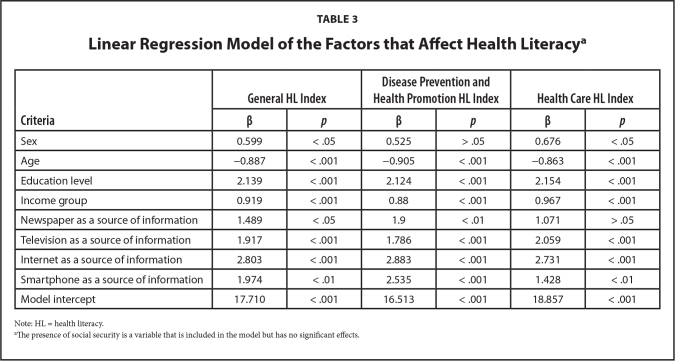
Linear Regression Model of the Factors that Affect Health Literacy^a^

**Criteria**	**General HL Index**		**Disease Prevention and** **Health Promotion HL Index**		**Health Care HL Index**	
	**β**	***p***	**β**	***p***	**β**	***p***
Sex	0.599	< .05	0.525	> .05	0.676	< .05
Age	−0.887	< .001	−0.905	< .001	−0.863	< .001
Education level	2.139	< .001	2.124	< .001	2.154	< .001
Income group	0.919	< .001	0.88	< .001	0.967	< .001
Newspaper as a source of information	1.489	< .05	1.9	< .01	1.071	> .05
Television as a source of information	1.917	< .001	1.786	< .001	2.059	< .001
Internet as a source of information	2.803	< .001	2.883	< .001	2.731	< .001
Smartphone as a source of information	1.974	< .01	2.535	< .001	1.428	< .01
Model intercept	17.710	< .001	16.513	< .001	18.857	< .001

Note: HL = health literacy.

a The presence of social security is a variable that is included in the model but has no significant effects.

## Discussion

It has been noted that HL should assess a person's skills as well as consider the interaction between the provision of health care services and people (Hernandez, 2009). The health promotion and health prevention domains that were separately evaluated in the conceptual framework of HLS-EU were addressed as a single domain in the THLS because in Turkey these domains are intertwined in terms of both the health care services provided as well as the social perception of health behavior. In addition, the World Health Organization (2018) states that disease prevention and health promotion have several common goals, and that there is a considerable overlap between these functions. We believe that HL can be more accurately measured when countries use scales tailored to the characteristics of their own society and health care systems.

### Health Literacy Level

This study shows the importance of population-based studies. According to our study, the proportions of people with inadequate and problematic HL in Turkey were 30.9% and 38%, respectively. Different HL measures can lead to different results; however, our scale is generally comparable to the HLS-EU scale. Thus, we think that we can compare our results with studies that use the HLS-EU scale. In the HLS-EU, 12.4% of the participants had inadequate HL, whereas 35.2% had problematic HL (Sørensen et al., 2015). The proportion of people with inadequate HL in Turkey is 30.9%, which is more than twice that of HLS-EU. According to the HLS-EU, Bulgaria (26.9%) and Austria (18.2%) had the highest proportion of people with inadequate HL (Sørensen et al., 2015), so Turkey scores worse than Bulgaria in terms of adequate HL. According to a study conducted in Japan using HLSEU-47, the ratios of respondents with inadequate and problematic HL were 49.9% and 35.5%, respectively (Nakayama et al., 2015). These results indicate that although limited HL is an important public health concern worldwide, its prevalence varies by country.

### Socioeconomic Determinants of Health Literacy

In the present study, higher age was associated with lower THLS score in all regression models. Studies have shown that advanced age is a risk factor for low HL (Kutner et al., 2006; Protheroe et al., 2017; Wu et al., 2017). Low HL may become more prevalent in the future for developing countries with a growing elderly population, such as in Turkey.

The regression model showed that men had an advantage in terms of the general and health care HL indices. Although some studies have reported that men have an advantage (Liu et al., 2015; Toci et al., 2014), others have reported that women have an advantage (Beauchamp et al., 2015). Certain studies identified no difference between the sexes (Eronen et al., 2019; Schaeffer et al., 2017; Wu et al., 2017). The varying relationship between HL and gender in different societies may be attributable to the fact that gender, in a social sense, is constructed by different societal conditions. Further studies may help reveal the different dimensions of the relationship between HL and sex.

Education level had a statistically significant effect on HL in all models. Different studies conducted worldwide also found that educational level is a determinant of HL (Beauchamp et al., 2015; Kutner et al., 2006; Liu et al., 2015; Protheroe et al., 2017; Toci et al., 2016; Wu et al., 2017). In the present study, higher HL was associated with higher income in all models. This is consistent with previous studies that have demonstrated that HL was higher in people with higher perceived financial status or household income (Bo et al., 2014; Eronen et al., 2019; Liu et al., 2015; Protheroe et al., 2017; Toci et al., 2016; Wu et al., 2017). The significant HL differences between the education and income groups show that HL is a reflection of social inequalities.

### Media Used as the Source of Health-Related Information and Health Literacy

According to the present study, the most frequently used medium as a source of health-related information was the internet (48.6%), followed by television (33%), newspapers (8.9%), and smartphones (6.5%). The frequency of use for the other sources was less than 5%. Studies indicate a high prevalence of using the internet as a source of health-related information. According to the National Trends Survey conducted in the United States, the leading source of health-related information was the internet (68.7%) (Swoboda et al., 2018). A study conducted in Hong Kong showed that 87.4% of the respondents used the internet to seek health-related information (Wong & Cheung, 2019).

All sources of health-related information had a significant effect on the model, with the only exception being the use of newspapers, which showed no significant effect in the health care HL domain. This illustrates the impact of the sources on shaping the level of HL. In our model, the internet had the highest impact on HL compared with other sources of information.

In a study based on the secondary analysis of the 2003 National Assessment of Adult Literacy (NAAL) data for people age 65 years and older, a regression model was created for the factors that determine HL (Cutilli et al., 2018). In this model, which included socioeconomic variables such as income, education, and ethnicity, a lower rate of using the internet (β = −5.231), books (β = −5.982), and magazines (β = −5.552) as a source of information resulted in decreased levels of HL. Newspapers, radio, or television did not exert a significant effect on the model (Cutilli et al., 2018). This study showed that the internet had a similar effect on the model as books and magazines. On the other hand, in our study, the effect of the internet on the model was higher than the other sources of information assessed. This difference may be attributable to the fact that the study based on NAAL data included people age 65 years and older. This comparison shows that different patterns can be observed in different age groups in terms of the sources of health-related information that have an impact on HL. On the other hand, a study using the English Longitudinal Study of Ageing data showed that after adjusting for cognitive decline and other covariates, consistent internet use was associated with lower HL (Kobayashi et al., 2015).

Various studies have shown that low HL is associated with less frequent use of the internet as a source of health-related information (Chakkalakal et al., 2014; Gutierrez et al., 2014; Levy et al., 2015). However, it should be noted that the quality of information provided on the internet remains questionable (McCray, 2005).

There are few studies that have investigated the relationship between HL and sources of health-related information other than the internet. In one study, the highest mean HL score was observed in those who reported that they used the internet, followed by those who reported that they used books and brochures as sources of health-related information (Lubetkin et al., 2015). As a determinant of HL, the South Korea National Survey provides an example of a parameter that is both a socioeconomic variable and a source of health-related information. The study showed that barriers to accessing information and expensive books and magazines were predictors of inadequate HL (Jeong & Kim, 2016). Another study showed that adequate HL was associated with owning a smartphone (Bailey et al., 2015).

The relationship between HL and sources of health-related information is occasionally conceptualized as a relationship in which the HL level is the predictor and the use of health-related information sources is the outcome (Kim & Utz, 2018). However, due to its multidimensional nature, HL may have a two-way relationship with health-related parameters, as it is observed with both health status and the use of health care services. On the other hand, when evaluating health-related information sources and determinants of HL, it is important to consider social inequalities. It is believed that there is a disparity between the existing literacy skills of the population and the required HL level. Studies show that those with the lowest levels of HL have the lowest access to health information. This situation has been conceptualized as the “inverse information law” (Rowlands & Nutbeam, 2013). In our study, the reported use of media as a source of health-related information was included in the model concerning the determinants of HL to demonstrate the influence of socioeconomic characteristics and the sources of health-related information on HL.

## Strengths and Limitations

The THLS scale consisted of questions based on self-report. Therefore, important skills, such as numeric HL, were not included in the scope of the assessment. This is an important limitation of using this study to compare rates between countries, as some differences may reflect cultural differences between societies for how people respond to such survey items.

On the other hand, self-reported scales are a more practical tool for national surveys involving a large number of participants. The majority of HL scales are self-assessment tools with objective scales, such as the Test of Functional Health Literacy in Adults and the Newest Vital Sign. It is has been shown that self-reported scales have low possibilities of eliciting stigma (Pleasant et al., 2019). A systematic review including articles that measured both performance-based and self-reported HL shows that most studies found no difference between performance-based and self-reported scales (Kiechle et al. 2015). Nevertheless, because HL includes different skills, self-reported questions cannot evaluate all of its components. Therefore, performance-based HL scales should be included as part of the national survey.

## Conclusion

In Turkey, differences in HL are associated with income, educational level, and age. These findings demonstrate that HL is linked to social inequalities. Variations in access to the sources of health-related information are associated with HL inequalities and constitute a suitable point of intervention against these inequalities. In particular, the internet could be proposed as a medium for such interventions, considering that it was found to have the highest impact on HL compared with the other sources. Together, identifying factors associated with HL and evaluating the effectiveness of intervention tools can lead to more successful outcomes. Based on our findings, creating internet resources with health information tailored to the older adults and people with lower education may help improve HL. Awareness of the risk for low HL in these groups also would be useful for informing those who produce internet content related to health information. Supporting people with low incomes by providing internet access could contribute to health promotion through improving HL. These types of interventions can help prevent the law of inverse information and support health equality.
